# Supplementary material for: Epstein–Barr Virus Promotes Oral Squamous Cell Carcinoma Stemness through the Warburg Effect
Source: Int J Mol Sci. 2023 Sep 14;24(18):14072. doi: 10.3390/ijms241814072 (PMC10531857; doi:10.3390/ijms241814072)
Supplement: Supplementary file 1 [file ijms-24-14072-s001.zip › ijms-2564485-supplementary.pdf]

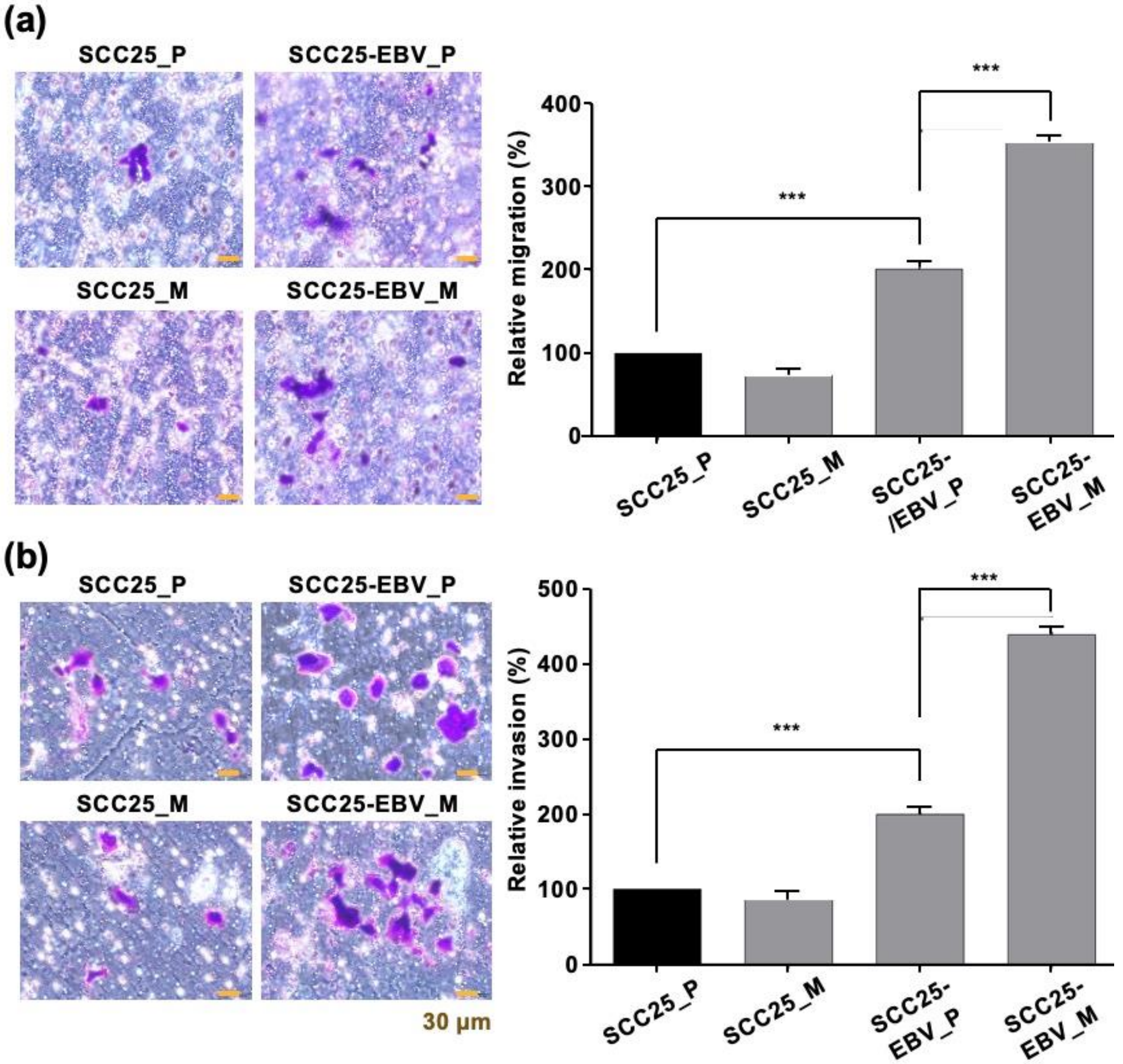

Figure S1. EBV promotes malignant phenotype of SCC25 cells after passage by xenograft model. Tumor cells were isolated from tumor tissues and examined for cell migration (a) and invasion (b) by transwell assay. P: parental cells and M: cells isolated from tumor tissues after passage by mice xenograft model. ***: $p < 0.001$.

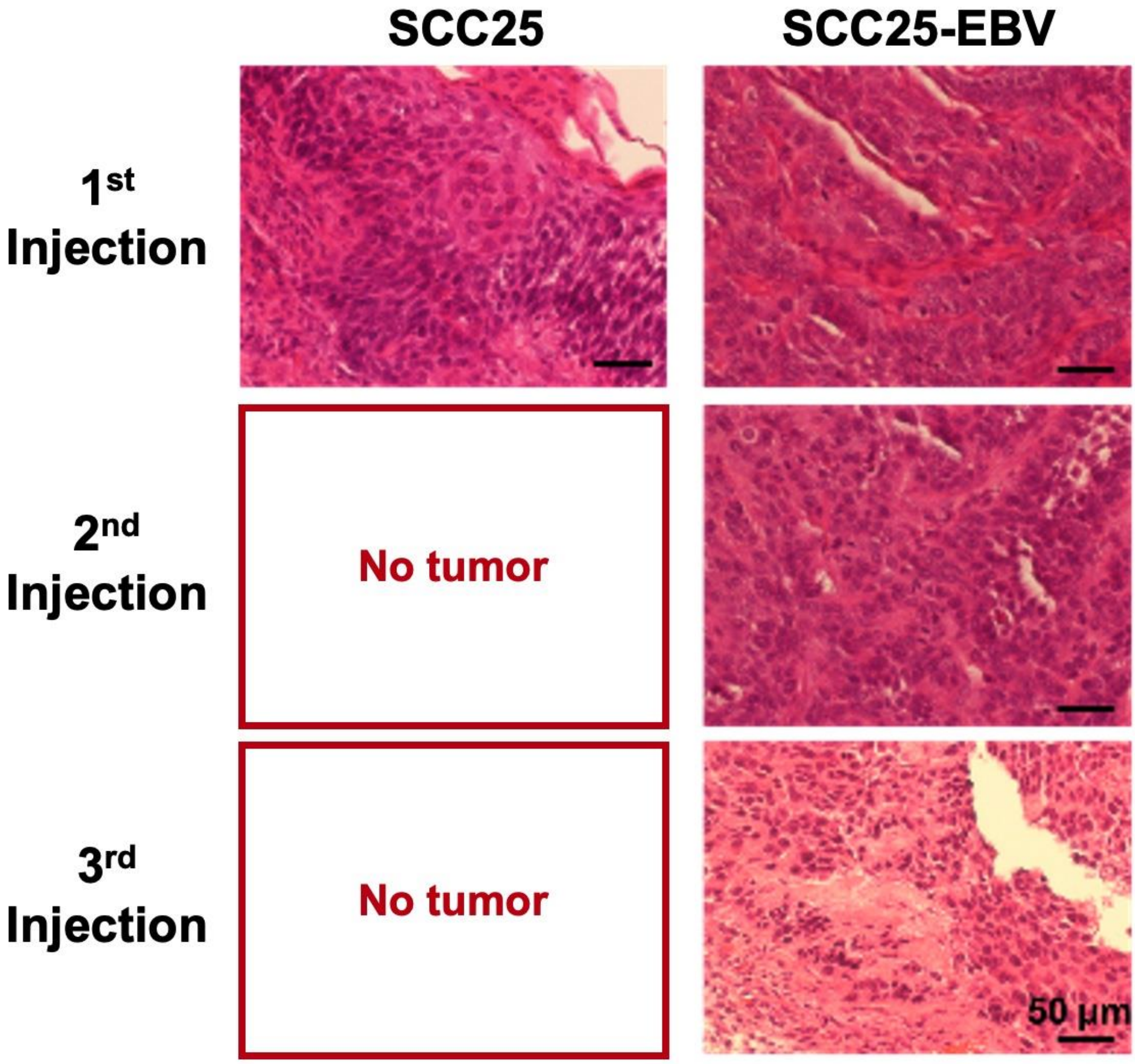

Figure S2. The histology of tumor tissues serially transferred by Balb/c mice as shown in Figure 7(a). Tumor tissues of SCC25 or SCC25-EBV were examined by H&E staining. Magification; 20X.

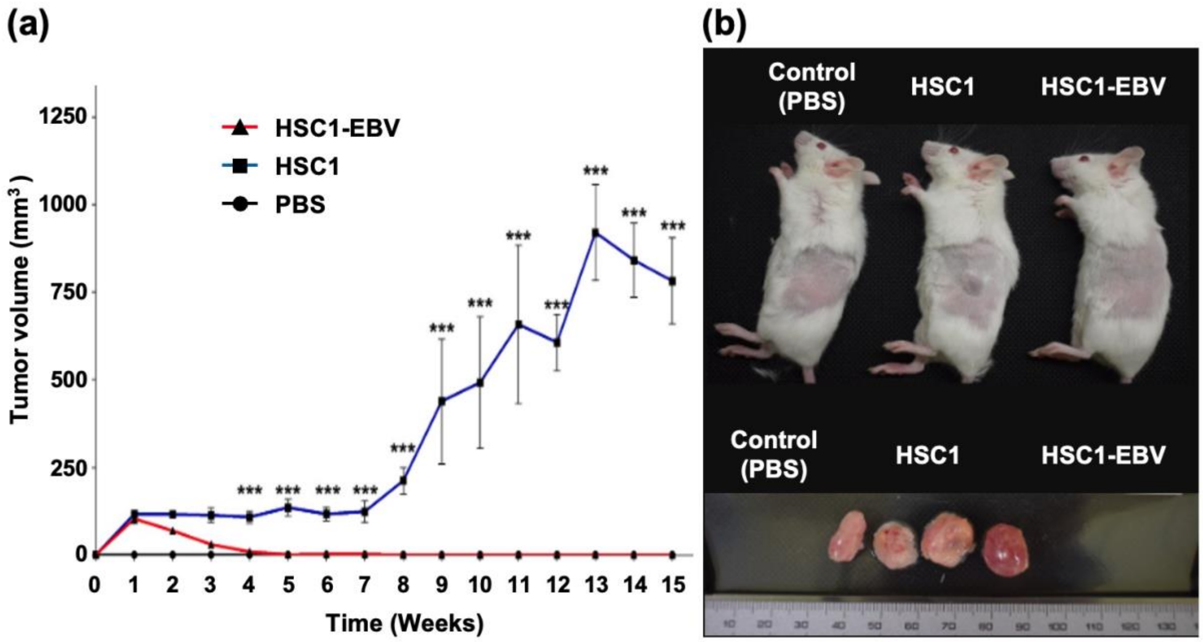

Figure S3. EBV infection did not promote tumor in HSC1 cells by the xenograft model. (a): HSC1 or HSC1-EBV cells were suspended with phosphate-buffered saline (PBS) and were subcutaneously injected into SCID mice. Four mice were injected with cells or PBS. Tumor volumes were measured weekly by caliper. (b): Upper panel shows tumors grown at the flank of mice. Lower panel shows each tumor excised from mice. *: $p < 0.05$.

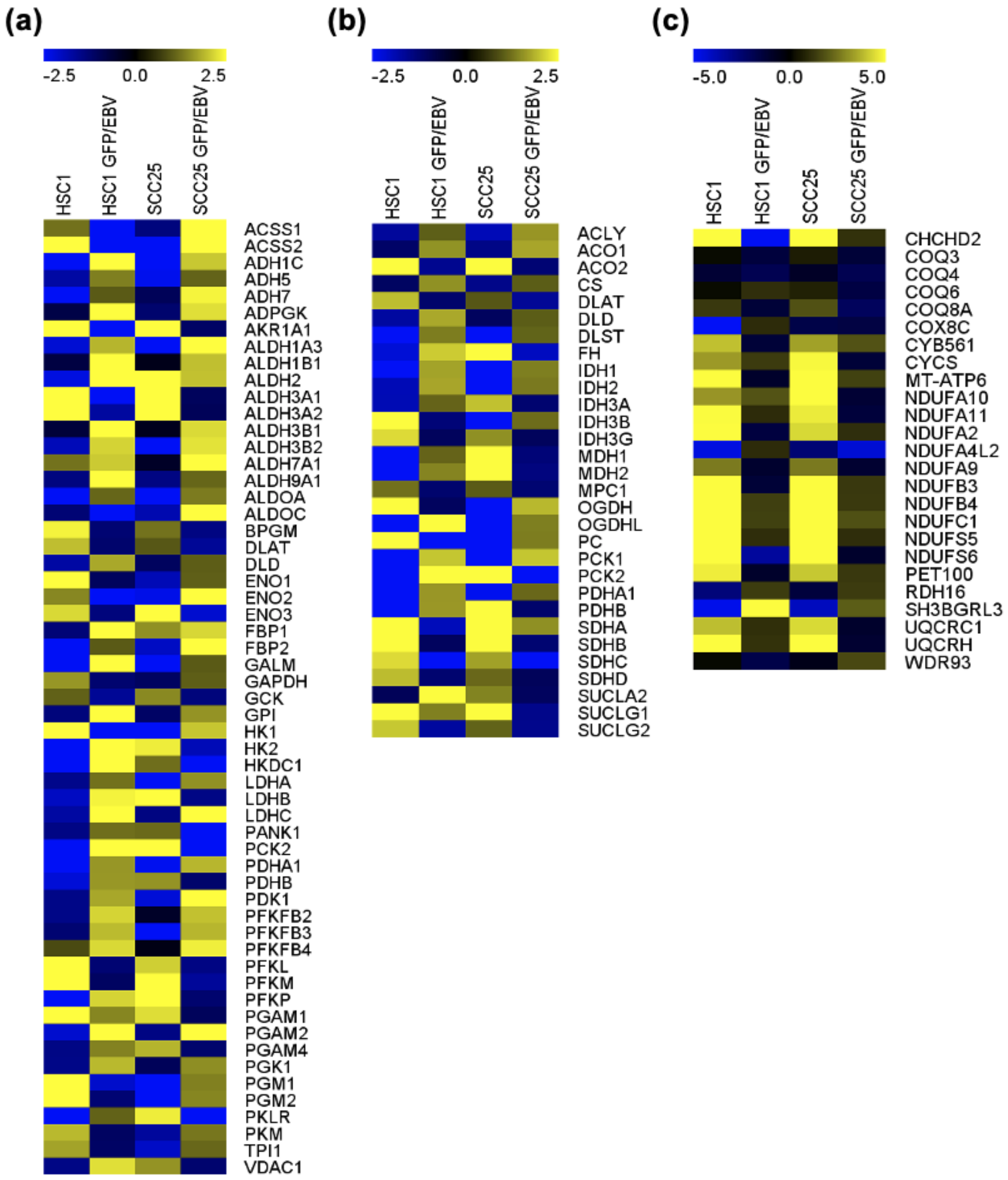

Figure S4. The expression levels of genes that were associated with glycolysis (a), TCA cycle (b) and electron transport chain (c) in EBV-positive and EBV-negative cell line quantified by microarray technique.

Table S1. Significantly up-regulated apoptosis-associated genes in HSC1-EBV cells.

Entrez gene ID	Gene name	Log2(FC)
9274	BAF chromatin remodeling complex subunit BCL7C (BCL7C)	4.31
581	BCL2 associated X, apoptosis regulator (BAX)	3.92
27113	BCL2 binding component 3 (BBC3)	4.07
666	BCL2 family apoptosis regulator BOK (BOK)	3.01
598	BCL2 like 1 (BCL2L1)	3.20
83596	BCL2 like 12 (BCL2L12)	2.10
440603	BCL2 like 15 (BCL2L15)	3.03
8837	CASP8 and FADD like apoptosis regulator (CFLAR)	3.33
1048	CEA cell adhesion molecule 5 (CEACAM5)	4.95
4680	CEA cell adhesion molecule 6 (CEACAM6)	5.60
10395	DLC1 Rho GTPase activating protein (DLC1)	2.60
220042	DNA damage induced apoptosis suppressor (DDIAS)	2.41
55332	DNA damage regulated autophagy modulator 1 (DRAM1)	2.23
54097	FAM3 metabolism regulating signaling molecule B (FAM3B)	2.02
23017	Fas apoptotic inhibitory molecule 2 (FAIM2)	2.02
355	Fas cell surface death receptor (FAS)	3.02
51454	GULP PTB domain containing engulfment adaptor 1 (GULP1)	7.59
3725	Jun proto-oncogene, AP-1 transcription factor subunit (JUN)	3.88
8462	KLF transcription factor 11 (KLF11)	3.24
4170	MCL1 apoptosis regulator, BCL2 family member (MCL1)	2.84
4193	MDM2 proto-oncogene (MDM2)	3.25
10783	NIMA related kinase 6 (NEK6)	2.89
91662	NLR family pyrin domain containing 12 (NLRP12)	3.77
4832	NME/NM23 nucleoside diphosphate kinase 3 (NME3)	3.54
10201	NME/NM23 nucleoside diphosphate kinase 6 (NME6)	2.11
5292	Pim-1 proto-oncogene, serine/threonine kinase (PIM1)	2.62
11040	Pim-2 proto-oncogene, serine/threonine kinase (PIM2)	3.48
415116	Pim-3 proto-oncogene, serine/threonine kinase (PIM3)	3.11
5899	RAS like proto-oncogene B (RALB)	3.72
6461	SH2 domain containing adaptor protein B (SHB)	2.92
30011	SH3 domain containing kinase binding protein 1 (SH3KBP1)	4.36
6881	TATA-box binding protein associated factor 10 (TAF10)	2.10
29844	TCF3 fusion partner (TFPT)	2.20
25816	TNF alpha induced protein 8 (TNFAIP8)	3.09
7188	TNF receptor associated factor 5 (TRAF5)	2.04
8797	TNF receptor superfamily member 10a (TNFRSF10A)	2.15
8795	TNF receptor superfamily member 10b (TNFRSF10B)	2.66
8793	TNF receptor superfamily member 10d (TNFRSF10D)	2.83
51330	TNF receptor superfamily member 12A (TNFRSF12A)	2.28
55504	TNF receptor superfamily member 19 (TNFRSF19)	3.13

3604	TNF receptor superfamily member 9 (TNFRSF9)	3.04
9966	TNF superfamily member 15 (TNFSF15)	7.55
51499	TP53 regulated inhibitor of apoptosis 1 (TRIAP1)	2.11
677	ZFP36 ring finger protein like 1 (ZFP36L1)	3.10
317	apoptotic peptidase activating factor 1 (APAF1)	2.87
8312	axin 1 (AXIN1)	3.27
79444	baculoviral IAP repeat containing 7 (BIRC7)	2.88
23705	cell adhesion molecule 1 (CADM1)	7.51
1116	chitinase 3 like 1 (CHI3L1)	6.56
64651	cysteine and serine rich nuclear protein 1 (CSRNP1)	3.41
26999	cytoplasmic FMR1 interacting protein 2 (CYFIP2)	2.72
1800	dipeptidase 1 (DPEP1)	2.26
1.00E+08	double homeobox 4 (DUX4)	3.16
10913	ectodysplasin A receptor (EDAR)	5.34
79767	engulfment and cell motility 3 (ELMO3)	3.54
2012	epithelial membrane protein 1 (EMP1)	2.31
2014	epithelial membrane protein 3 (EMP3)	3.37
3956	galectin 1 (LGALS1)	3.81
284110	gasdermin A (GSDMA)	4.44
51022	glutaredoxin 2 (GLRX2)	2.93
3002	granzyme B (GZMB)	10.25
2999	granzyme H (GZMH)	2.78
1647	growth arrest and DNA damage inducible alpha (GADD45A)	2.39
4616	growth arrest and DNA damage inducible beta (GADD45B)	2.91
9026	huntingtin interacting protein 1 related (HIP1R)	2.29
3635	inositol polyphosphate-5-phosphatase D (INPP5D)	4.93
3689	integrin subunit beta 2 (ITGB2)	3.10
3429	interferon alpha inducible protein 27 (IFI27)	2.61
2537	interferon alpha inducible protein 6 (IFI6)	2.93
11009	interleukin 24 (IL24)	5.00
79960	jade family PHD finger 1 (JADE1)	3.83
23095	kinesin family member 1B (KIF1B)	3.41
4853	notch receptor 2 (NOTCH2)	2.82
3164	nuclear receptor subfamily 4 group A member 1 (NR4A1)	4.45
5058	p21 (RAC1) activated kinase 1 (PAK1)	2.94
55367	p53-induced death domain protein 1 (PIDD1)	2.83
5551	perforin 1(PRF1)	5.34
5366	phorbol-12-myristate-13-acetate-induced protein 1 (PMAIP1)	2.94
7262	pleckstrin homology like domain family A member 2 (PHLDA2)	2.33
1263	polo like kinase 3 (PLK3)	2.92
5047	progestagen associated endometrial protein (PAEP)	7.01
84306	programmed cell death 2 like (PDCD2L)	2.97

10015	programmed cell death 6 interacting protein (PDCD6IP)	3.55
8682	proliferation and apoptosis adaptor protein 15 (PEA15)	2.27
23645	protein phosphatase 1 regulatory subunit 15A (PPP1R15A)	3.64
5794	protein tyrosine phosphatase receptor type H (PTPRH)	3.69
388	ras homolog family member B (RHOB)	3.08
84236	rhomboid domain containing 1 (RHBDD1)	2.97
55312	riboflavin kinase (RFK)	3.33
6197	ribosomal protein S6 kinase A3 (RPS6KA3)	3.38
117584	ring finger and FYVE like domain containing E3 ubiquitin protein ligase (RFFL)	3.41
54476	ring finger protein 216 (RNF216)	3.08
9262	serine/threonine kinase 17b (STK17B)	2.53
30061	solute carrier family 40 member 1 (SLC40A1)	2.93
10011	steroid receptor RNA activator 1 (SRA1)	2.48
84951	tensin 4 (TNS4)	3.87
7048	transforming growth factor beta receptor 2 (TGFBR2)	3.94
84260	trichoplein keratin filament binding (TCHP)	3.93
8565	tyrosyl-tRNA synthetase 1 (YARS1)	2.86
7429	villin 1 (VIL1)	9.40
64393	zinc finger matrin-type 3 (ZMAT3)	2.86
